# KVFinder: steered identification of protein cavities as a PyMOL plugin

**DOI:** 10.1186/1471-2105-15-197

**Published:** 2014-06-17

**Authors:** Saulo HP Oliveira, Felipe AN Ferraz, Rodrigo V Honorato, José Xavier-Neto, Tiago JP Sobreira, Paulo SL de Oliveira

**Affiliations:** 1 National Laboratory of Biosciences, P.O. Box 6192, CEP 13083-970 Campinas, SP, Brazil; 2 Inter-institutional Grad Program on Bioinformatics, University of São Paulo, Rua do Matão, 1010 IME-USP, Bloco B, sala 137, CEP 05508-050 São Paulo, Brazil; 3 System Approaches to Biomedical Sciences Industrial Doctorate Centre - Doctoral Training Centre, University of Oxford, 127, Magdalen Road, Postal code OX4 1RJ Oxford, UK

**Keywords:** KVFinder, Protein cavities, Volume calculation, PyMOL plugin

## Abstract

**Background:**

The characterization of protein binding sites is a major challenge in computational biology. Proteins interact with a wide variety of molecules and understanding of such complex interactions is essential to gain deeper knowledge of protein function. Shape complementarity is known to be important in determining protein-ligand interactions. Furthermore, these protein structural features have been shown to be useful in assisting medicinal chemists during lead discovery and optimization.

**Results:**

We developed KVFinder, a highly versatile and easy-to-use tool for cavity prospection and spatial characterization. KVFinder is a geometry-based method that has an innovative customization of the search space. This feature provides the possibility of cavity segmentation, which alongside with the large set of customizable parameters, allows detailed cavity analyses. Although the main focus of KVFinder is the steered prospection of cavities, we tested it against a benchmark dataset of 198 known drug targets in order to validate our software and compare it with some of the largely accepted methods. Using the one click mode, we performed better than most of the other methods, staying behind only of hybrid prospection methods. When using just one of KVFinder’s customizable features, we were able to outperform all other compared methods. KVFinder is also user friendly, as it is available as a PyMOL plugin, or command-line version.

**Conclusion:**

KVFinder presents novel usability features, granting full customizable and highly detailed cavity prospection on proteins, alongside with a friendly graphical interface. KVFinder is freely available on http://lnbio.cnpem.br/bioinformatics/main/software/.

## Background

Proteins perform their biological functions mainly by interacting with other molecules, ranging from small ions to macromolecules such as proteins, and/or nucleic acids. Experience demonstrates that interactions between protein binding sites and their ligands depend on the physical properties displayed at contact interfaces [1]. These interactions are highly specific and variable across specific protein domain and ligand classes, thus constraining the efficient interaction of a given protein to a few types of ligands. Such strong specificities result from a high level of spatial complementarity between binding sites and ligands [2].

The development of computational methods to predict and characterize binding sites in proteins has been an active research theme, which can be demonstrated by the large number of theoretical methods developed for this purpose. The published algorithms can be classified into three distinct categories, as they can be based on geometry, on energy, or on evolutionary principles. Geometry-based methods locate cavities by analyzing the molecular surface, generally using a 3D grid, spheres or tessellation techniques, and comprise a majority of available software. Examples include LIGSITE [3], CAST [4], SURFNET [5], PASS [6], SCREEN [7], POCASA [8], PocketPicker [9], Fpocket [10], POCKET [11], CavitySearch [12], DogSite [13], TRAPP [14], PHECON [15] and VOIDOO [16].

Energy-based methods identify cavities by analyzing the energetic interaction between the target protein and a probe, usually represented by a chemical group. Examples of this approach are GRID [17], CS-Map [18], DrugSite [19], QsiteFinder [20] and PocketFinder [21]. Methods based on evolutionary principles rely on the search for conserved residues in sequence alignments and information of known active site profiles. Examples of algorithms using this approach are ConSurf [22] and Rate4Site [23]. Meta-servers that combine more than one approach have also been published, such as MetaPocket [24], FINDSITE [25] and LigSiteCSC [26].

Geometry-based methods present some advantages when compared to other approaches. In contrast to evolutionary approaches, geometry-based methods do not rely on prior knowledge, thus being independent on the number of available sequences. Geometry-based methods are also more straightforward than energy-based methods, which are highly dependent of force field parameterization and scoring functions.

Here we introduce KVFinder, a geometrical grid-based method, which presents some distinguished capabilities, such as search space segmentation, a user friendly interface and full customizable parameters, able to identify and analyze different kinds of protein cavities, including pockets, tunnels and shallow crevices.

The parameter customization is designed to solve some of the major flaws of geometry-based methods. Grid-based methods are sensitive to grid spacing, but in KVFinder this is a user defined parameter, which, combined with the space segmentation capability, creates the opportunity to generate fine high-resolution representation of cavities. Another common problem in geometry methods is the definition of cavity ceiling, which in our method can be directly controlled by customizable probe sizes. KVFinder’s space segmentation capability creates multiple possibilities for cavity analysis because it allows the study of relevant subpockets and the assignment of its individual characteristics, e.g., sub-sites in enzymes or protein kinases, joint cofactor and substrate binding site.

Finally, one special concern on this project is usability, as we noted that many of the available methods fail on this aspect. KVFinder has basic and advanced usage modes, and is available not only on the command line (which is better suited for quick, or high-throughput analyses), but also as a PyMOL Plugin [27] with a user friendly GUI for Linux and Windows.

## Implementation

### Finding cavities

**Figure 1:**
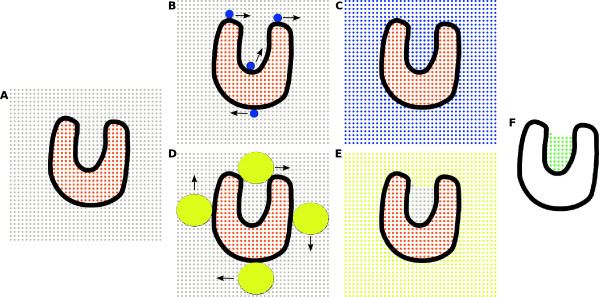
**Didactic representation of KVFinder algorithm. A)** Two dimensional representation of a protein cavity inserted in a grid. Orange points represent the area occupied by the protein, gray points stand for empty space. **B)** The blue Probe In rolling around the protein. **C)** After the Probe In prospects the protein, all grid points overlapped by the probe are marked in blue. **D)** The Probe Out (yellow) also prospects the protein. **E)** Points overlapped by the Probe Out are marked in yellow. **F)** The Cavity is defined as the difference between points explored by the two probes.

The first challenge for any cavity detection method lays on the cavity definition itself. There is a marked discrepancy on the way different methods define the limits and the ceiling of cavities and there seems to be no clear rationale or a formal definition of a protein cavity. In KVFinder we employ a geometrical cavity definition that is based on the theory of mathematical morphology [28-30].The prospected protein is inserted in a 3D grid, in which the points can be either occupied by the protein, when they lay within the Van der Waals radii of any of the protein atoms, or empty. In KVFinder, the Van der Waals radii are user-defined, through a configurable dictionary file. Screening of the empty points is made using two probes, defining two molecular surfaces of the protein. The probe is centered at each empty grid point and defines a surface comprised of overlapped and not-overlapped points. Overlapped points must be inside the probe radius and away from any protein atom by more than the Van der Waals radii. A small probe, dubbed Probe In, is meant to reach empty space in the protein, thus delineating the intern part of the cavities. The same process is repeated with a big probe, the Probe Out, generating another surface of points. The Probe Out has restricted access to the empty space within the protein, generating a surface used to define the border between the cavity and the bulk. Those two surfaces are combined in order to extract the protein cavities: overlapped grid points on the Probe In surface that are not overlapped on the Probe Out surface are considered as cavity points. A didactic representation of this process is exposed on Figure 1.

Once cavity points are defined on the grid, the next step separates points forming different cavities. Cavity points are considered as belonging to the same cavity only when there is spatial connectivity between them. For this step, we use a recursive implementation of the DFS algorithm [31]. Considering a cavity point to be a node, we define an accessible node to be searched as a cavity point that is on the same row, column or diagonal of our current node, with grid coordinate difference no greater than one. A new search is made for every cavity point that has not yet been marked as belonging to a cavity. Every point visited recursively during a search procedure is marked with the same label, which marks it as belonging to the same cavity.

For each cavity found, KVFinder performs a spatial characterization based on its grid points. Cavity volume is calculated as the sum of grid sized cubes (voxels) comprised in the cavity space. The surface area is computed by summing grid sized squares formed by grid points on the cavity surface. KVFinder is equipped with a user-defined volume threshold, which suppresses any cavity under a given volume.

Every grid-based method is sensitive to grid spacing. Higher density grids enable richer spatial representation, which are useful for detailed study of cavities, but imply higher computational costs. To overcome this issue, KVFinder makes grid spacing an input parameter, enabling the user to explore a balance between performance and precision.

Given KVFinder's definition of a cavity as the space between molecular surfaces defined by two probes, it is no surprise that probe size has a major impact on the results. The Probe In is preset at 1.40 Å, the approximate radius of a water molecule, defining a solvent accessible surface. Therefore the interior of the calculated cavity is not consisted of any empty space within the protein. It is only defined by the accessible parts to molecules of a certain size. A varied Probe In size can be used for solvents other than water. However, the setup of an optimal radius for the Probe Out is not a straightforward step, as it may vary substantially depending on characteristics of the analyzed protein. This probe defines a ceiling for the cavity. Thus, if the Probe Out is small enough to enter the cavity, it will make the defined cavity shallow. Depending on how deep this Probe Out can roll into the cavity, it might even make the cavity disappear. On the other hand, if the big probe is very large, it can demand more computational time.

To establish an optimal value for the ceiling probe we performed a simulation using 198 known drug targets [32], screening different values for the probe size. The simulation consisted of making a cavity search using whole protein mode and varying the Probe Out sizes between 2 Å to 8 Å in increments of 0.5 Å steps. A prediction is considered correct when the center of mass of the cavity is within 4 Å of any ligand atom [26]. Cavities were ranked based on cavity volume and the top three cavities were analyzed. To evaluate the ability of KVFinder to work as a cavity detection software, we used the same benchmark dataset above and compared the results to other methods [32].

### Space segmentation

A new feature introduced by KVFinder is the space segmentation, which means that the prospected region can be user defined or considering the whole protein. With this feature, the user is able to split the cavity in subpockets, generating a spatial characterization of separate parts of the cavity. By introducing the usability of the search space restriction, KVFinder creates a new set of possibilities for more detailed cavity analysis. Detailed space definition can be a valuable asset on ligand binding studies, because it allows a detailed analysis of the space occupied by different parts of the ligand. The space segmentation also addresses the problem of resolution sensitivity, which affects all grid-based cavity search methods. By restricting the search around a given area of interest, a higher resolution representation can be achieved at a much lower computational cost. The search space is defined by an interactive box, created by the PyMOL Plugin.

### User interface

**Figure 2:**
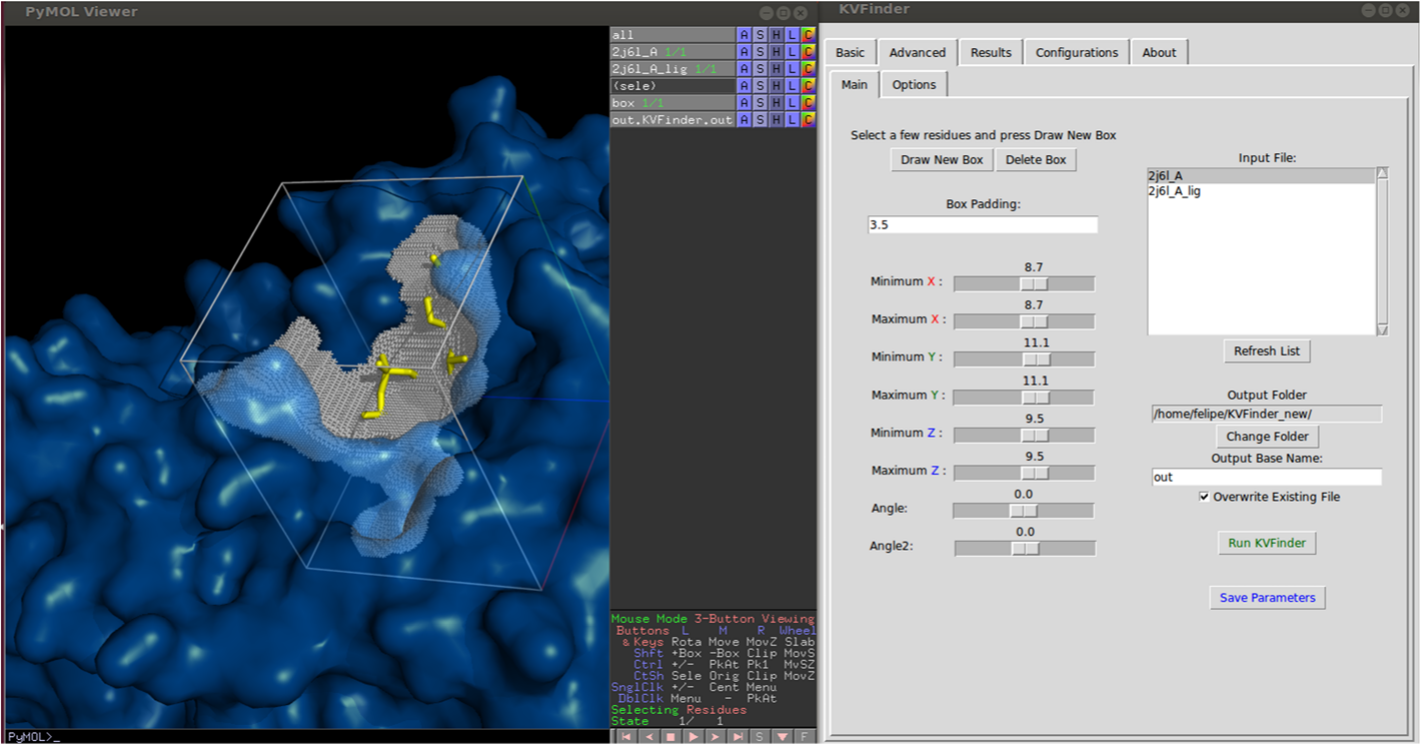
**Overview of KVFinder GUI.** Example of KVFinder’s graphical user interface. The search space delimitation is made through a customizable box. In the example, it was defined around the ligand of the [PBB: 2J6L].

**Figure 3:**
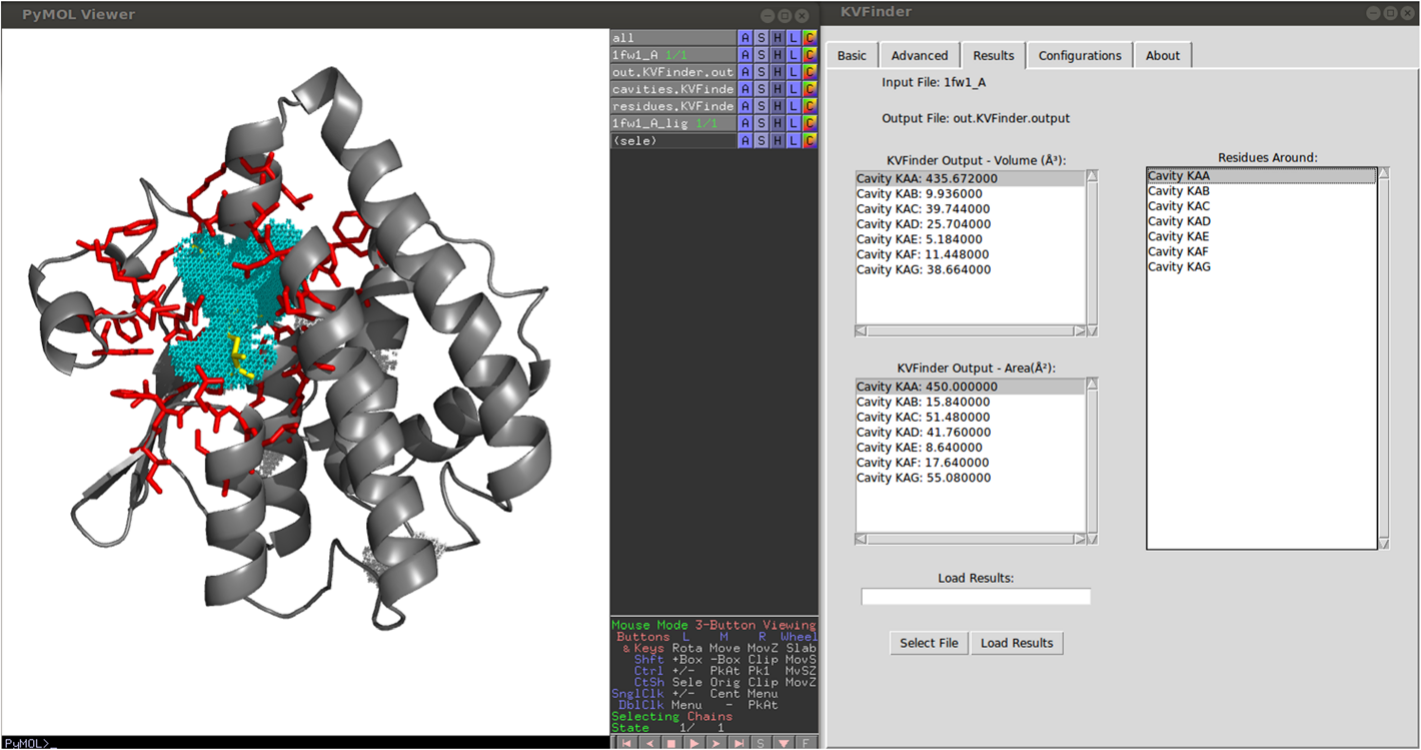
**KVFinder results on the GUI.** Example of results being displayed on the graphical interface. Here a whole protein prospection was made on the target protein structure [PDB:1FW1], and information of every cavity is displayed. To aid cavity identification, a highlight tool is available through selection. We can also see interactively the forming residues of each cavity.

Usability was one of the major concerns during KVFinder development. Alongside with a set of customizable parameters, KVFinder brings an innovative, easy to use, graphical interface implemented as a PyMOL Plugin. The interface has a basic mode tab, the one click analysis, which displays a limited group of options to set up small and big probe sizes, grid spacing and volume filter.In the advanced mode the user can access the space segmentation feature. First the search space is defined through a graphical box, which is built around the default PyMOL selection. The graphical box is fully customizable, both in size and in position. This feature allows the user a free choice of the search space, enabling the use of previous knowledge (Figure 2). Beyond the basic parameters of size of small and big probes, grid spacing and volume filter, the advanced mode presents three additional options. Users can define the inner limit of the cavity in two ways: 1) as molecular surface, limited by the Van der Walls radii of atoms; 2) as the solvent accessible surface (SAS) defined by the passage of a smaller probe. A desirable capability of our software is limiting the cavity prospection around a ligand. Depending on the parameters defined, number of grid points generated might be too large, which demands a longer processing time. KVFinder addresses this offset by means of a step re-dimension option. This option rescales the grid spacing, granting fast analysis, but should be turned off for refined high resolution results.In both basic and advanced modes KVFinder outputs a list of all detected cavities along with their respective volumes and areas, as well as the amino acid residues that contribute to cavity formation. All this information is accessible interactively in the results tab, as shown in Figure 3. The output data is also available in text files, containing information about the detected cavities and a PDB file containing the cavity points which can be used in any molecular visualization software. Furthermore, KVFinder also works as a command line program, presenting all the options available on the interface mode.

## Results and discussion

### The effect of the probe out size

**Figure 4:**
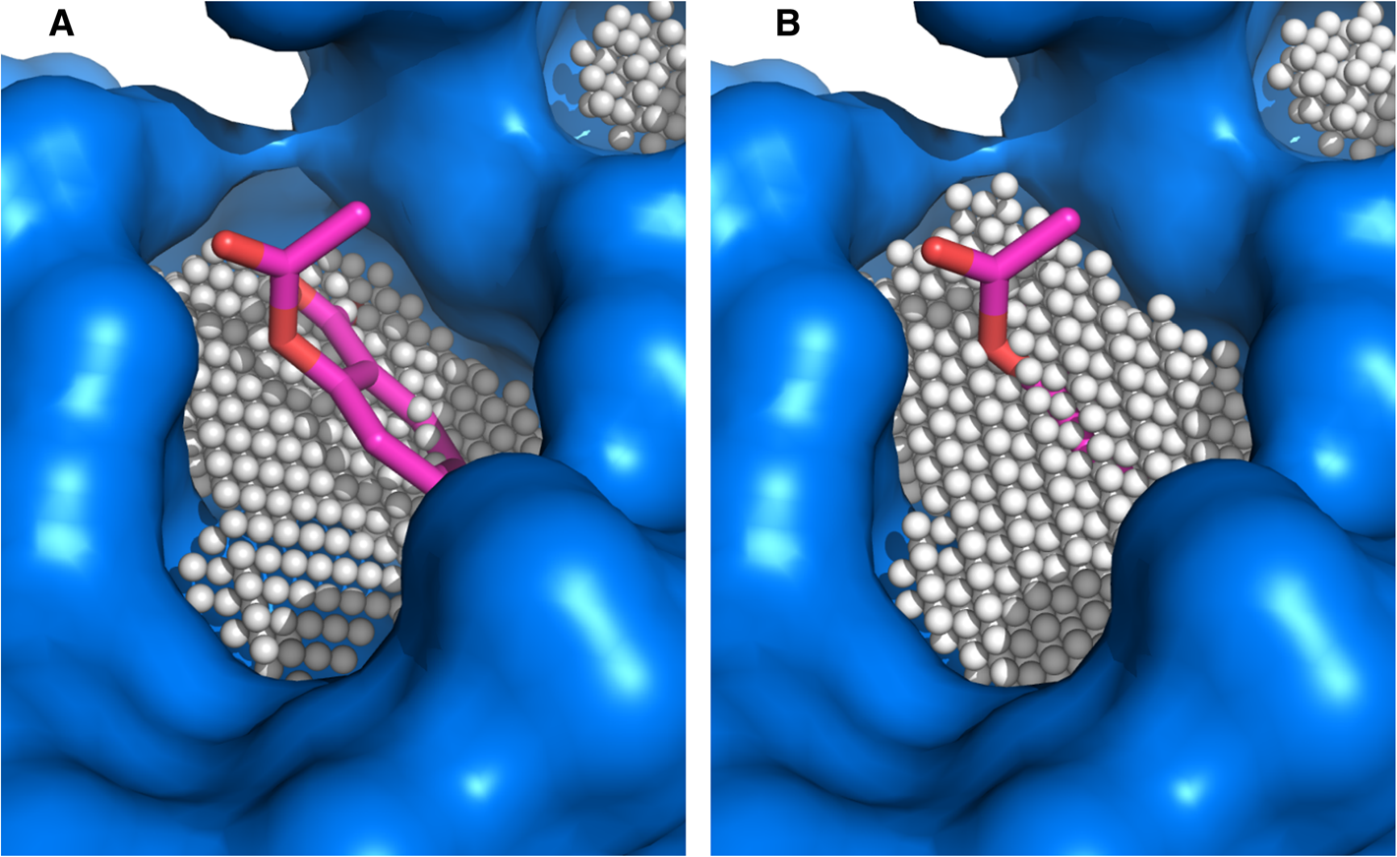
**Cavity ceiling definition. A)** Prospection of the binding site on the target protein structure [PDB:1OXR], using a 3 Å Probe Out, which defines a shallow cavity that barely touches the ligand. **B)** The same cavity prospected with 6 Å Probe Out, defining a cavity that covers most of the ligand.

**Figure 5:**
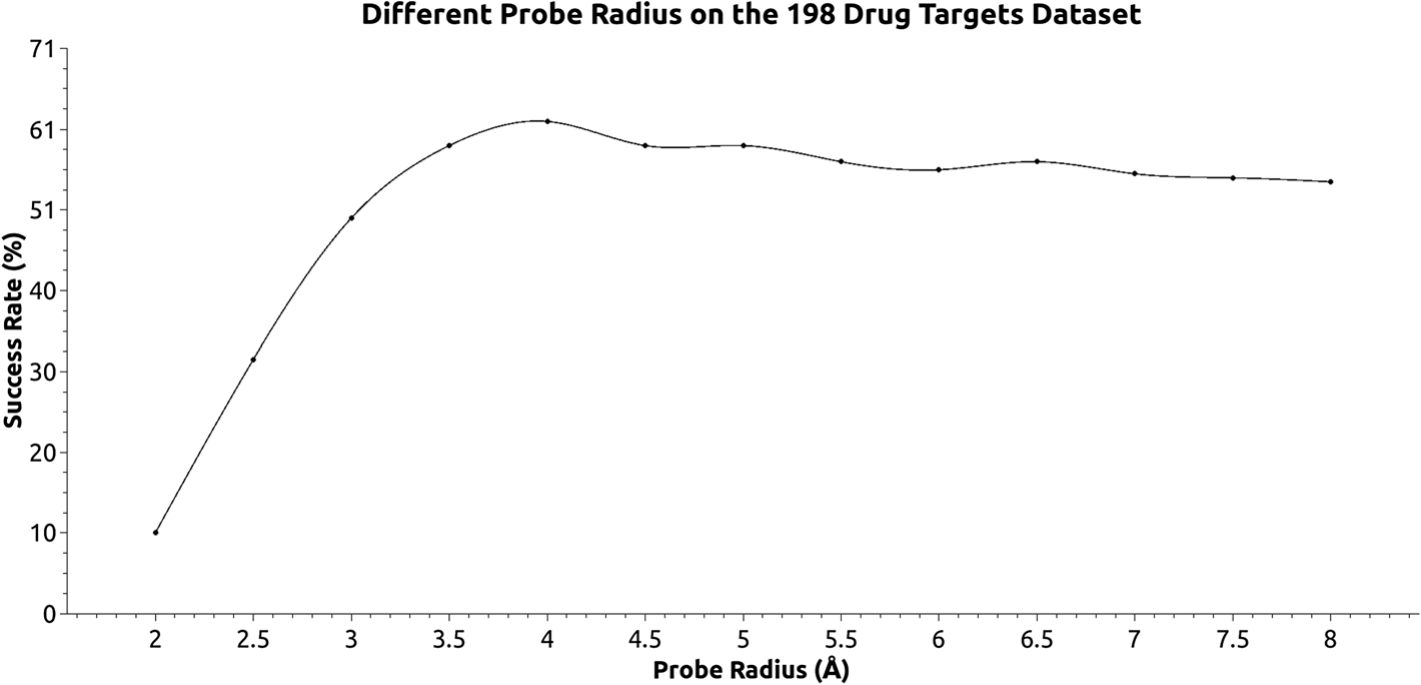
**Effect of using different outer prober on the benchmark test.** Success rate evaluation for different Probe Out sizes on the 198 known drug targets dataset. Smaller probes achieve a low success rate, as it tends to define shallow cavities, with a higher probability of losing cavities. Bigger probes demands a higher computational cost and cannot surpass the success rate achieved between 3.5 Å and 4 Å.

KVFinder is designed to be a steered cavity prospection tool. Our method works with fully customizable parameters and can be adjusted according to the user's needs. Grid spacing and Probe In radius are preset, facilitating the use of our software. Defining a standard value for the Probe Out is not an easy task, as its size is heavily correlated with the kind of cavity prospected. The effect on the cavity volume and shape of different big probe sizes can be viewed in Figure 4. In order to define a radius value for the Probe Out better suitable for the majority of cases, we evaluated KVFinder’s success rate using a benchmark dataset of 198 known drug targets.Our results demonstrated that the Probe Out radius has a major influence on the success rate (Figure 5). The preset value for this parameter was set as 4.0 Å, which presented a 62% overall success rate. It is important to emphasize that the Probe Out size, that optimized the overall success rate for this particular dataset, can be used as a starting point for a generic problem. However, this parameter can and must be changed to fit to specific protein analysis. When analyzing shallow and wide crevices, such as found in the substrate binding sites of protein kinases, it would require a larger probe.

### Comparing KVFinder to other methods

To validate KVFinder as a cavity detection software, we compared its results to other well-known methods. This test was conducted using the same benchmark dataset used to evaluate the effect of the probe out size. We evaluated the success rate in three cases, considering just the top cavity, the top 2 and top 3 cavities. Data of the same test using other methods was extracted from literature [32]. Using default parameters, KVFinder had the highest success rate among individual software on the top 1 and top 3 tests (Table 1), falling behind only after MetaPocket 2.0, which combines results of prediction mechanisms of several software. We performed a second test using KVFinder’s Probe Out customization, ranging from 2 Å to 8 Å with an increment of 0.5 Å, and combining these predictions. Using this combined prediction, KVFinder was able to outperform all the other methods, achieving 76% success rate on the top 3 test.

**Table 1:** Success rate of binding site prediction of different software for 198 known drug targets structures

**Method**	**Top 1**	**Top 2**	**Top 3**
**KVFinder Combined Prediction**	**67**	**74**	**76**
MetaPocket 2.0 [24]	61	70	74
**KVFinder**	**51**	**56**	**62**
LIGSITECS [26]	48	57	61
PASS [6]	35	50	56
Q-SiteFinder [20]	40	54	62
SURFNET [5]	24	30	34
GHECOM [33]	39	51	56
ConCavity [34]	47	53	56
Fpocket [10]	31	48	57
POCASA [8]	43	54	56

### Space segmentation

**Figure 6:**
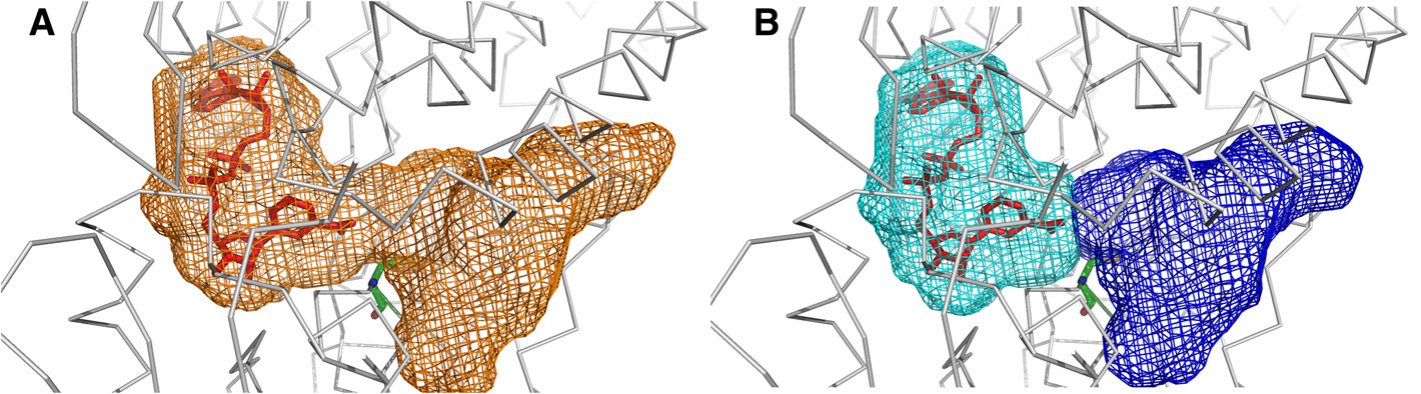
**Space segmentation of ALDH1 channel. A)** Analysis of the substrate access channel ALDH1 [PDB:1BXS]. The catalytic cysteine (Cys 302) is highlighted in stick representation. **B)** Space segmentation of the substrate access channel, substrate binding site is represented in blue and the NAD binding site in cyan. Volume determination of this second subpocket is essential, as its size is directly correlated with the protein functionality [35].

A great part of KVFinder flexibility comes from using the mathematical morphology approach and leaving its crucial elements as customizable parameters such as the grid spacing and the probe size. A key and innovative feature introduced here is the space segmentation, an asset for studies in which there is a need to explore specific sections of cavities. Although an automatized approach for cavity segmentation is possible [13] we apply an innovative steered segmentation approach, as there are many cases in which a specific biological knowledge is mandatory. To demonstrate our space segmentation feature, we analyzed the substrate entry channel of the enzyme aldehyde dehydrogenase 1 (ALDH1) [PDB:1BXS]. Previous studies proposed that the overall geometry of the substrate entry channel (SEC) determines ALDH1/2 specificity [35]. As shown in Figure 6, the functional tunnel is composed of two portions, the NAD and the substrate access site. The authors in [35] established 'hot spots' for the specificity of ALDH1/2 in three residues (124, 459 and 303; human ALDH2 numbering). To show the ability of KFinder in correctly identify the substrate entry channel of ALDH1, we defined the search space limiting the grid point analysis in a cutting plane through C-alpha of Cys303 and the catalytic residue Cys302, well-known to form the bottom of the SEC. This feature endows the user full control of how to visualize and segment a cavity of interest.

## Conclusion

KVFinder provides an efficient geometrical characterization of protein cavities. On the blind site prospection test, it achieved a 76% success rate, outperforming other methods. KVFinder's main focus is the innovative steered search approach, relying on a large set of customizable parameters, making possible complex and detailed analyses of cavities. The user can split the cavity in subpockets, define the cavity ceiling, adapt the output to match the protein topology and adjust the spatial representation resolution. All these features are accessible through an easy to use graphical interface. KVFinder is a powerful asset that provides needed tools to gain a deeper understanding on protein cavities.

## Availability and requirements

**Project name:** KVFinder

**Project home page:**http://lnbio.cnpem.br/bioinformatics/main/software/

**Operating system(s)**: Ubuntu 12.04, Windows 7 SP 1

**Programming language:** C, C++, Python/Tk

**Other requirements:** PyMOL v.1.4.1. on Linux, PyMOL v.1.3 in Windows

**License:** GPLv3

## Abbreviations

ALDH1: Aldehyde dehydrogenase 1; SEC: Substrate entry channel; DFS: Depth-first search.

## Competing interests

The authors have declared that no competing interests exist.

## Authors’ contributions

FANF and SHPO developed the algorithm, RVH and FANF wrote the manuscript, JXN and TJPS discussed the theory and helped with the manuscript, PSLO coordinated the project. All authors read and approved the final manuscript.
